# Télésanté en contexte de pandémie et de déconfinement : pratiques infirmières innovantes et partenariats pour des communautés équitables, sécuritaires et durables

**DOI:** 10.1177/1757975920980720

**Published:** 2021-03

**Authors:** Judith Lapierre, Sylvain Croteau, Marie-Pierre Gagnon, Jacques Caillouette, Fanny Robichaud, Suzanne Bouchard, José Côté, Iris Aboulhouda, Karel Ménard, Sabrina Picard, Ève-Marie Myette, Vicky Drapeau, Bilkis Vissandjée, Brigitte Kankindi, Christina Doré

**Affiliations:** 1 Université Laval, Québec, Canada; 2 Centre intégré de santé et de services sociaux de l'Outaouais du Québec, Gatineau, Québec, Canada; 3 Université de Sherbrooke, Sherbrooke, Québec, Canada; 4 Université du Québec en Outaouais, Québec, Canada; 5 Faculté des sciences infirmiéres, Université de Montréal, Montréal, Québec, Canada; 6 Mimosa du Quartier, Gatineau, Québec, Canada; 7 Université du Québec en Abitibi Témiscamingue, Rouyn-Noranda, Québec, Canada

**Keywords:** logement communautaire, télésanté, justice sociale, enquête, santé communautaire, pandémie, diversité culturelle, pratiques infirmières

## Abstract

La télésanté connait un essor fulgurant en ce contexte de pandémie. Or, en cette période d’insécurité mondiale, la santé préventive reprend ses droits. En période de déconfinement, la discipline et la cohésion sociale peuvent se relâcher. Cette enquête sociale vise à décrire un programme d'intervention à distance, réalisé en partenariat avec des locataires de logement communautaire, des infirmières et des étudiantes pour soutenir la littératie en santé au temps de la COVID-19. Le *Programme de déconfinement en toute sécurité* vise à renforcer les mesures préventives et de soutien avec des groupes en contexte de vulnérabilités économiques et sociales à l’aide de la télésanté. Les infirmières ont développé des pratiques cliniques et psychosociales et renforcé la littératie en santé, soutenant les mesures de santé publique post-COVID-19, surveillant l’éclosion de nouveaux foyers et apaisant les souffrances issues du confinement. À l’aide de cibles de performance des systèmes de santé et d’une perspective de justice sociale, nous avons documenté les défis, les leviers et les menaces à l’usage des pratiques à distance en prévention. Vecteur d’une approche intégrée, la télésanté préventive peut cibler simultanément, la lutte contre les maladies non transmissibles et transmissibles et les inégalités. La pandémie de COVID-19 renvoie à un nouvel équilibre des enjeux qui exige un accompagnement et des pratiques de santé communautaire engagées et critiques.

## Introduction

Urgence sanitaire mondiale, la pandémie de COVID-19 a aussi entraîné un ralentissement économique qui touche d’autant plus les communautés qui vivent en contexte de vulnérabilités économiques et avec des emplois précaires. Cinq constats liés à la COVID-19 et aux inégalités qu’elle entraîne nous interpellent. Premièrement, les femmes monoparentales, travaillant plus souvent dans des secteurs affectés par la pandémie, sont les principales victimes de la crise économique mondiale (1). Deuxièmement, leurs apports, leurs connaissances et leurs perspectives ont été peu documentés dans les stratégies visant à améliorer la préparation et la réponse à la COVID-19 (2). Troisièmement, la littératie en santé est fondamentale dans la lutte contre la COVID-19 (3). Quatrièmement, la fracture dans les réseaux d’aide et dans les filets de sécurité sociale change le sens et l’expérience du confinement et de la distanciation physique (3), entraînant notamment une hausse du sentiment d’insécurité et des préoccupations chez 33 % de la population canadienne (4). Les effets néfastes sur la santé mentale de la population sont nombreux, diversifiés et peuvent perdurer (5). Cinquièmement, l’usage des médias numériques a été un moyen de communication privilégié pour les populations confinées et isolées. Or, une proportion significative de la population planétaire (plus de 40 %) est exclue du monde numérique, avec un accès limité à Internet ou encore un faible niveau de littératie numérique (6). Les enjeux d’équité sont donc accrus, notamment auprès de certains groupes. Renforcer les compétences des citoyens en littératie numérique en santé est une des solutions envisagées pour contrer la désinformation qui circule sur les médias sociaux (7). Ces constats nous amènent à optimiser le développement de nouvelles formes de connectivité précipitées par la COVID-19 afin de prévenir l’exclusion, réduire les écarts entre les classes et limiter des disparités liées à l’ethnicité et au genre. Notre intervention de télésanté vise à renforcer le pouvoir d’agir des personnes frappées plus durement par les effets délétères de la COVID-19. Elle contribue au renforcement des capacités de sociabilité, de participation citoyenne et de solidarité. Considérant que les inégalités raciales ont déjà frappé en matière de taux d’infection, d’hospitalisations et de décès, notamment aux États-Unis (8,9), les communautés les plus défavorisées socioéconomiquement subiraient de manière disproportionnée les effets négatifs de la COVID-19 (10). De plus, au moment où le logement est la première protection contre le coronavirus, la stabilité résidentielle est devenue une question de vie ou de mort (11,12). Par ailleurs, une revue Cochrane récente (13) indique que les infirmières composent le groupe professionnel le plus enclin à intervenir en éducation à la santé et dans le soutien à l’autogestion. Sachant que l’engagement communautaire est crucial pour mettre un terme à la menace que pose le nouveau coronavirus (14) et que la télésanté a connu un essor fulgurant dans ce contexte de pandémie (15), nous proposons un partenariat novateur afin d’apporter une réponse rapide à la COVID-19.

## Programme

Le *Programme de déconfinement en toute sécurité par la télésanté préventive* (*Programme*) s’est déroulé du 14 mai au 26 juin 2020. La télésanté réfère à toute activité ou service, pratiqués à distance, au moyen des technologies de l'information et des communications qui facilitent les autosoins, le suivi et le traitement (16,17). La télésanté préventive comprenait un appel d’évaluation clinique initiale portant sur le profil de santé, l’évaluation des symptômes, l’évaluation sociale, l’évaluation du risque nécessitant un dépistage et l’élaboration d’un plan d’intervention en soins infirmiers, d’environ une heure, ainsi que des appels de suivis hebdomadaires plus brefs (questionnaire de suivi). Cinq étudiantes en sciences infirmières (stagiaires) de l’Université Laval ont prêté main forte à l’infirmière de l’organisme communautaire, afin d’assurer la mise en œuvre du programme auprès des 19 familles participantes. Les évaluations cliniques initiales et les questionnaires de suivi étaient saisis sur SEKMED, plateforme informatisée d’appui à la démarche clinique incluant une base de connaissances évolutives en santé (cliniques, expérientielles, probantes). L’immédiateté, la capacité de réponse rapide, la reconnaissance des mots dans le système accélérant les associations aux documents cliniques recherchés, en a fait la plateforme idéale. Une communauté virtuelle de pratique sécurisée sur le site québécois Passerelles, *Pratiques de télésanté préventive* s’est ajoutée pour échanger sur les suivis cliniques, les plans d’intervention et de surveillance ainsi que pour héberger des documents utiles. Les données ont été compilées sur REDCAP à l’Université Laval. Le *Programme* adopte une approche globale de prévention avec des activités connexes aux suivis de télésanté, sur les déterminants (cuisine solidaire, espace informationnel, distribution de masques, distributeurs de gels antibactériens). L’organisme de bienfaisance a reçu un fonds d’urgence de Centraide pour ces activités connexes. De plus, une organisation a financé des cartes cadeaux aux locataires ayant perdu leur emploi durant la COVID-19 pour contrer les risques d’insécurité alimentaire.

## But et objectifs spécifiques

Après un mois et demi de confinement, le Québec amorçait en mai 2020, un virage progressif vers le déconfinement. Le *Programme*, auprès de personnes qui vivent en logement communautaire, visait à renforcer les mesures préventives liées à la COVID-19 et de soutien, le pouvoir d’agir et la dignité.

**Trois objectifs scientifiques** sont documentés:

1) Décrire l’évolution des symptômes liés à la COVID-19 et celle de certains déterminants de santé;
2) Décrire la satisfaction des familles monoparentales du *Programme*;
3) Décrire les retombées initiales.

## Méthode

### Devis

Une enquête sociale (18) a permis de décrire un programme de télésanté préventive réalisé en partenariat avec le secteur de l’habitation communautaire. Les fondements théoriques du programme et de son évaluation comprennent, entre autres, la perspective de justice sociale (19). Fraser propose une conception tridimensionnelle de la justice sociale intégrant la reconnaissance, la redistribution et la parité de participation. Elle propose une redistribution équitable des ressources et des opportunités, ce que favorise le *Programme*. L’accès à des savoirs communs sur la pandémie et personnalisés à l’aide de conseils brefs jumelé à la participation des femmes à des échanges sur leur vécu conduisent à une forme de reconnaissance de leur identité et de leur dignité. La redistribution des savoirs, mise sur le levier du collectif pour développer des forces collectives, briser l’isolement et créer l’appartenance, qui à son tour peut stimuler la participation au sein des instances décisionnelles, opérationnelles et politiques du milieu de vie. Le programme de télésanté préventive stimule la participation citoyenne pour la santé, développe la littératie numérique des femmes et construit les mailles du filet de sécurité sociale en renforçant la cohésion sociale. À cette théorie, nous avons ajouté des cibles du cadre de performance des systèmes de santé (20), soit la dimension de l’accessibilité et celle de la qualité (efficacité, sécurité, réactivité) qui ont également guidé le développement du *Programme* et son évaluation ainsi que l’analyse environnementale de la télésanté (SWOT) (21). Diverses sources de données ont été croisées : les questionnaires de suivis de télésanté saisis dans SEKMED, l’entrevue qualitative réalisée à mi-parcours, le sondage quantitatif de fin de projet complété en ligne ainsi qu’un groupe focalisé avec les infirmières. Une approbation éthique du Comité d’éthique de la recherche avec des êtres humains de l’Université Laval a été obtenue.

#### Analyses

Des tests du khi-deux exact ont été effectués sur les données issues des questionnaires répétés de suivis pour vérifier la présence d’associations entre les variables catégorielles mesurées une seule fois pendant le suivi. Une régression logistique estimée par modèle d’équations d’estimations généralisées a permis de tester l’effet linéaire du temps sur la présence de stress lié à la pandémie. Ces résultats sont présentés avec un rapport de cotes (RC) et son intervalle de confiance à 95 %. Les données qualitatives de l’entrevue mi-parcours ainsi que du groupe focalisé ont fait l’objet d’une analyse de contenu traditionnelle des verbatims incluant la codification des segments, l’identification de thèmes récurrents et la réduction en catégories.

### Participants

Les locataires ont été invités à participer au projet de télésanté et à l’étude associée. L’habitation est située au cœur du centre-ville de la quatrième plus grande ville en importance du Québec au Canada. Neuf pourcent de la population de cette ville est constituée de personnes immigrantes. Plusieurs indicateurs de l’état de santé de la population sont moins favorables que dans le reste de la province (22). Dix-neuf locataires sur 21 ont accepté de participer à l’étude. L’organisme sans but lucratif, membre d’un regroupement régional des habitations communautaires, a accepté de participer à l’étude; il mettait en place des mesures exceptionnelles en période de déconfinement avec l’équipe clinique et de recherche et l’occasion de documenter l’implantation et les perceptions des locataires était accueillie très favorablement.

## Résultats

### Données sociodémographiques

La moyenne d’âge des 19 participants est de 36 ans (20 ans et 52 ans), la majorité s’est identifiée comme des femmes (18 femmes, 1 homme) et la plupart sont des femmes issues de la diversité culturelle, avec un parcours migratoire. Trois sources de revenu sont présentes parmi les locataires : aide financière de dernier recours pour 8 personnes, salaire minimum pour 5 personnes, emploi stable permanent pour 6 personnes. Sept participants ont reçu un fonds supplémentaire lié à la COVID-19, du gouvernement fédéral du Canada et 12 n’ont rien reçu, parce qu’ils n’étaient pas éligibles. Dix-sept participants ont des enfants à charge. Selon les facteurs de risques connus de la COVID-19, quatre femmes ont des atteintes respiratoires, l’une est considérée comme immunosupprimée et trois autres font de l’hypertension artérielle diagnostiquée.

### Évolution des symptômes de la COVID-19 avec le déconfinement

Les symptômes suivants associés à la COVID-19 ont été recensés : fièvre (5 %), myalgie (11 %), rhinorrhée (5 %), dyspnée (5 %), douleurs thoraciques (5 %), toux (16 %), expectorations (11 %), diarrhée (5 %), fatigue augmentée (16 %), confusion (5 %) et perte d’équilibre (5 %). Il est à noter que trois participantes (16 %) ont réalisé un test de dépistage. Deux personnes ont été testées aux deux semaines parce qu’elles étaient préposées en milieux de soins et une, pour la présence des symptômes qui perdurent dans le temps.

### Stress et caractéristiques des participantes

Quatorze femmes (74 %) affirment avoir ressenti, au moins une fois, un stress face à la pandémie. Le pourcentage de présence de stress spécifique en lien à la pandémie a été comparé selon différents facteurs : le changement de revenu (avec perte 88 % vs sans perte 55 %, *p* = 0,18), le nombre d’enfants (de 100 % avec 4 enfants à 33 % avec aucun enfant, *p* = 0,24), le niveau de risque (moyen/élevé 67 % vs faible 69 %, *p* = 1,00). La présence de différents symptômes pendant la période de suivi (*p* > 0,05) ou le changement de poids (*p* = 1,00) ne sont pas non plus liés au stress. Le gain de poids a atteint entre 15 et 20 livres (7 à 9 kg) chez certaines femmes.

### Revenu

Au niveau de l’impact sur le revenu du ménage, six participantes (32 %) disent avoir eu une diminution de leur revenu face aux fermetures des différents milieux de travail. Toutefois, une femme (5 %) mentionne avoir vu son revenu augmenter, grâce à l’aide financière fournie par le gouvernement.

### Stress et déconfinement

Les données du *Programme* ont permis de conclure que le stress s’atténue avec le temps chez les participantes. En effet, lors de l’évaluation initiale des symptômes et des déterminants, 13 d’entre elles (68 %) confirmaient ressentir un stress spécifique en lien avec la pandémie. Ce pourcentage diminue ensuite au fil des semaines, jusqu’à atteindre 8 % (*p* = 0,000 7 ; RC = 0,58 [0,41–0,81]), soit une seule personne, après six semaines de déconfinement.

### Suivi des symptômes cliniques

Dans le sondage auto-rapporté, 36,8 % (*N* = 7/19) des familles ont affirmé avoir été testées pour la COVID-19 durant le déconfinement. Cependant, 79 % (*N* = 15/19) rapportent ne pas avoir développé de symptômes durant la même période et aucun des enfants n’aurait développé de symptômes selon les parents.

### Satisfaction et expérience des familles à mi-parcours

- **Appréciation globale.** Les locataires ont mentionné apprécier la disponibilité, la réactivité et la flexibilité dans les horaires.

Les appels réguliers ont suscité une appréciation du soutien qui s’étend au-delà des informations sur les symptômes. Le seul fait de veiller sur les locataires et de les questionner sur leur bien-être, de les écouter, d’aborder les enjeux de stress, de sécurité et les impacts financiers sur l’alimentation du ménage, a été apprécié par les locataires. « On s’est senti accompagné … » « Quelqu’un se soucie de vous, cela monte le moral, cela rassure ». Le renfort par les stagiaires a entraîné un dynamisme dans le milieu. Le renforcement des mesures lors des appels de suivi était apprécié de même que les communications dédiées, dans l’espace informationnel à l’entrée de l’immeuble. « Les consignes affichées à l’entrée nous rappellent que la pandémie n’est pas finie et qu'elle est présente ». Finalement, une femme a fait valoir que la communication simple et directe avec la direction était appréciée : « j’apprécie la façon simple de communiquer avec les administrateurs de l’organisme et sans protocole ».

- **Utilité.** Près de 80 % des locataires ont perçu l’utilité du *Programme*. Cela repose surtout sur la possibilité de parler, de maintenir le moral, d’assurer un sentiment de sécurité, d’appeler les infirmières et même de contribuer à la formation des étudiantes. Les informations transmises ont été jugées utiles à 89,9 % soit pour 16 répondants.
- **Formule de télésanté**. À la question, est-ce un bon moyen que d’utiliser le téléphone, 89,9 % (*N* = 16/19) ont répondu oui.

Une locataire mentionne que « ce n’est pas toujours évident de se déplacer, avec les enfants quand on est mère monoparentale, alors que quand c'est par téléphone c'est plus facile, ça permet aux parents de gérer, on peut être au téléphone et faire autre chose en même temps avec les enfants ». Tous les locataires n’ont pas la haute vitesse ou un accès à Internet. Fortement appréciés, les appels de suivi étaient réalisés en fonction des disponibilités et précédés par un message texte pour convenir du meilleur moment pour l’appel.

- **Proactivité/réactivité.** Le *Programme* a permis aux familles d’évoluer dans un milieu qu’elles considéraient comme proactif et réactif. « L’organisme fait les efforts pour prendre soin des gens ». « On a des gens qui sont à l'écoute de nos inquiétudes et qui essayent de nous aider ».
- **Littératie en santé**. Quatre-vingt-trois pourcents des locataires (*N* = 15/19) ont affirmé avoir reçu des conseils lors des appels.

Concernant la compréhension des informations reçues, 100 % ont affirmé avoir compris les consignes. Le *Programme* a été perçu par les locataires, sauf une personne, comme étant personnalisé. Les familles se sentaient à l’aise et écoutées. « On est à jour avec l'information, donc quand tu as toute l'information, tu peux prendre des décisions éduquées la … ça aide beaucoup, ça empêche l'anxiété et de se sentir toute seule ». « On a reçu beaucoup de conseils sur les consignes du COVID-19 ». Renforcer les connaissances des signes et symptômes, cibler les informations selon les contextes familiaux et soutenir les mesures de sécurité ont été réalisés par les infirmières. « Oui, j’ai reçu des conseils et j’en ai tiré des leçons sur les mesures de sécurité, l'importance du port de masque, de distanciation sociale, de l’alimentation diabétique ». En plus, près de 50 % des locataires ont profité des appels pour poser d’autres questions relatives à leur santé.

- **Préoccupations et stress engendrés.** Les préoccupations des familles concernent l’incertitude et la peur continue de contracter la COVID-19 (*N* = 6/19). « Il y a une peur qui est permanente parce qu'on ne sait pas toujours où et quand on peut attraper le COVID-19 et le déconfinement contribue à accentuer cette peur ». Pour certaines familles, il était difficile d’occuper les enfants pendant le confinement. Le retour précoce à la garderie, la proximité entre les locataires, les changements dans les consignes et la réouverture des lieux publics étaient des sources d’inquiétudes avec le fait qu’il n’y ait pas de vaccin contre la COVID-19. Pour les travailleurs de milieux à risque, le risque de contamination plus élevé apportait son lot de stress. Le *Programme* a permis d’apaiser les stress et les préoccupations et a été perçu très aidant (9 personnes, 47,1 %) à extrêmement aidant (3 personnes, 17,6 %) pour un score combiné d’appréciation à 64,7 % contre moyennement aidant (7 personnes, 35,3 %).
- **Sécurité.** Aucun cas de COVID n’a été déclaré. Trois éléments menacent la sécurité. D’abord, il y a les visiteurs sur les lieux qui ne respectent pas les consignes. « Avec le déconfinement ce n'est plus contrôlé, y a plus de barrières ». « Ils représentent toujours un risque pour nous autres ». Puis, des itinérants occupent parfois les lieux entrant par des accès laissés libres par les locataires. Finalement, la distanciation physique reste un défi de taille avec les enfants dans les aires de jeux.
- **Isolement.** La diminution de contacts augmente le sentiment de sécurité personnelle. De plus, les familles ont décidé de ne plus partager les jeux extérieurs. Une minorité affirme ne sortir de leur logement que pour des choses essentielles comme aller à l’épicerie.
- **Équité**. L’identité culturelle semble faire partie du *vivre ensemble* qui se réalise dans une relative harmonie générale où la prise en compte de cette identité est rapportée par plusieurs. « Ici, il y a 80 % de population immigrante. Je me sens très respectée, les gens sont très ouverts, les gens sont compréhensifs ». « Chaque personne a sa propre religion, il y a un respect mutuel, il n'y a pas de chicane. Tout le monde respecte les autres ». Le *Programme* a aussi été jugé très respectueux de l’identité culturelle des locataires.

### Pratiques infirmières

Des pratiques infirmières d’évaluation, d’accompagnement, de conseils, de surveillance et de référencement ont été exercées. Des plans thérapeutiques infirmiers ont été élaborés et mis à jour. Prise de poids, stress (*lié à l’encadrement des enfants, au changement de routine, aux difficultés financières*), anxiété, manque de connaissances, faible réseau et difficultés financières sont quelques-uns des constats les plus fréquents. Les interventions les plus courantes ont ciblé le renforcement des consignes, les conseils nutritionnels et aussi l’assiduité au traitement médical des maladies chroniques préexistantes. Des enseignements ont été réalisés (symptômes, facteurs de risque et mesures préventives), des formations offertes (diabète, gestion de stress, prise de tension artérielle) ainsi que des conseils brefs sur l’importance du maintien d’un rythme d’activité physique. Quelques références ont été faites auprès d’autres ressources communautaires.

### Retombées de la télésanté perçue par les familles à la fin du projet

Toutes les familles sondées (100 %, *N* = 19/19) ont rapporté être satisfaites du *Programme* et la pertinence a été évaluée majoritairement favorable en combinant les scores tout à fait d’accord (57,9 %), d’accord (21,1 %) et plutôt d’accord (10,5 %). Jugée facile à utiliser par tous, avec un accueil par les infirmières évalué positivement, la stratégie apparaît souhaitable durant la période de déconfinement par l’ensemble (*tout à fait d’accord* à 52,6 % / *d’accord* à 36,8 % / *plutôt d’accord* à 10,5 %). La flexibilité et les accommodements ont facilité son usage pour la très grande majorité (90 %). La télésanté est pertinente selon les scores combinés en accord pour 94,7 % des familles (*N* = 18/19) pour toutes sortes d’autres services préventifs. Le fait d’être chez soi et d’avoir un accès à distance a été apprécié par l’ensemble, sauf une personne. À l’affirmation suivante, *la télésanté peut répondre à plusieurs de mes besoins de santé préventive et de dépistage précoce*, 100 % des répondants ont déclaré être en accord (*tout à fait d'accord* à 42.1 %, *d’accord* à 36.8 % *et plutôt d'accord* à 21.1 %). Il ressort également que 79 % (*N* = 15/19) des familles ont affirmé être en accord avec l’affirmation « J’ai obtenu des conseils que je vais utiliser » contre 3 personnes indifférentes et une personne en désaccord. Le fait d’être à distance n'a pas empêché de créer un lien avec l'intervenante qu’elles ne connaissaient pas pour 94,5 % (*N* = 18/19) des participants (*tout à fait d'accord* à 55,6 %, *d'accord* à 16,7 %, p*lutôt d'accord à* 22,2 %). Suite à l’affirmation : *Mon sentiment de sécurité a été renforcé par la télésanté*, les personnes sondées sont en accord à plus de 80 % (tout à fait d'accord à 44,4 %, d'accord à 38,9 %) et trois personnes sont indifférentes. Par ailleurs, la télésanté a aidé les personnes à respecter les consignes de santé publique pour 84,2 % des répondantes (*N* = 16/19). Finalement, à l’affirmation suivante : *Si la télésanté est disponible dans mon milieu de vie, je l'utiliserai*, les familles ont répondu globalement à l’affirmative (*tout à fait d'accord* à 42,1 %, *d'accord* à 31,6 %, *plutôt d'accord* à 21,1 %).

## Discussion

### Accessibilité et qualité du service

Certaines caractéristiques liées à l’accessibilité des services (*accessibilité et équité d’accès*) et celles touchant la qualité des services (*efficacité, sécurité, réactivité et continuité*) ont été explorées. L’équité d’accès désigne la capacité de fournir les soins et les services en fonction des besoins et sans égard aux caractéristiques personnelles. Le *Programme* a atteint ses objectifs d’accessibilité, la perception d’équité dans le *Programme* était grande. L’efficacité du service se mesure par la capacité à améliorer la santé et le bien-être. Les familles n’ont pas développé la COVID-19 et ont diminué leur niveau de stress durant le déconfinement, ce qui pourrait cependant être expliqué par le déconfinement progressif. Un autre devis de recherche serait nécessaire pour attribuer la causalité aux effets du *Programme*. Des analyses statistiques ont été effectuées malgré la faible taille d’échantillon, et aucune analyse de puissance n’a été réalisée. Ces résultats sont fournis à titre informatif, doivent être interprétés avec prudence, et une plus grande étude serait souhaitable afin d’en valider les conclusions. La structure du *Programme* et son opérationnalisation ont renforcé le sentiment de sécurité des familles. La réactivité ou la capacité de s’adapter aux attentes, aux valeurs et aux droits des personnes se sont avérées positives. Une très grande majorité de locataires a apprécié la capacité de l’organisme à protéger les familles et à répondre à leurs préoccupations. La continuité par une approche intégrée et coordonnée s’est construite par la concertation efficace de l’équipe clinique. Une synergie dynamique et proactive s’est rapidement installée autour des cibles visées.

### Télésanté et relation soignante

Selon une étude récente (23), 80 % des résidents de la région où s’est déroulée l’étude détiennent un téléphone intelligent, ce qui est plus élevé que dans l’ensemble du Québec (76 %). Par ailleurs, dans cette région, 92 % des foyers ont une connexion internet à domicile, comparativement à 93 % dans l’ensemble du Québec. L’usage du téléphone s’est cependant avéré la meilleure stratégie. Comme dans une autre étude récente auprès d’un groupe vulnérable (24), les familles ont préféré l’usage du téléphone à une autre forme de communication. Au plan de l’équité, de récentes données (25) indiquent l’importance du lien de confiance pour diminuer les risques de mauvaise pratique de télésanté. De même, la satisfaction, la disponibilité et l’importance accordée à la sécurité seraient tous des éléments favorables aux bonnes pratiques. La relation soignante s’est développée même avec la télésanté, qui a renforcé l’accès à des informations et le sentiment d’autogestion. Outillées et mieux informées, les familles ont amélioré leurs capacités décisionnelles et leur autodétermination. Une mère affirme **«** Je pense que vous ne pouvez pas résoudre les problèmes non plus, vous êtes là à l’écoute pour nous informer et nous outiller, je pense que la responsabilité revient à nous aussi ».

### Solidarité et réciprocité

Malgré la vigilance accrue, voire une certaine méfiance du voisinage, un esprit de solidarité s’est fait ressentir à travers les discours des locataires, par exemple les propos sur les besoins des uns et des autres et l’expression d’un « nous » collectif faisant référence aux locataires et à l’administration. Le *Programme* a fait naître une réciprocité, entre les locataires, entre les locataires et l’administration ainsi qu’entre les locataires et les étudiantes infirmières. Le stress était un facteur significatif de l’expérience des familles et comme dans l’étude de Moring (26), il est possible que des formes de détresse expérimentée évoluent vers l’expérience de stress post-traumatique, mais ce risque peut être limité par l’accès à des informations fiables et à un soutien continu.

### Durabilité

Plusieurs locataires ont souhaité voir le *Programme* être prolongé jusqu’à la fin de la pandémie, voire même d’envisager les appels sur une base régulière. Ce sont surtout les familles immigrantes, sans famille élargie à proximité, qui ont le plus largement apprécié le *Programme*.

### Justice sociale et pratiques de soins

La justice sociale requiert à la fois la redistribution et la reconnaissance, la notion de parité de participation en constituant le pivot (19). L’étude a contribué à des rapprochements sociaux, à renforcer la dignité, à faire reconnaître l’identité culturelle et à stimuler la participation citoyenne pour prévenir la transmission communautaire et éviter des éclosions dans le milieu de vie. L’exercice d’une pratique infirmière d’évaluation, d’accompagnement, de conseils, de surveillance et de références en situation réelle de crise sanitaire, autour d’enjeux de maladies transmissibles et non transmissibles (27,28), constitue une occasion très riche pour le développement de stratégies d’équité.

### Perspectives

S’intéresser aux défis des parents et des enfants avec des stress liés au confinement et à la distanciation sociale (29) est nécessaire. La transformation de l’enfance et de la parentalité mérite des analyses plus poussées autour des capacités d’adaptation et de la santé mentale (30). Les habitudes de vie ont été modifiées et les expériences auto-rapportées semblent indiquer des impacts négatifs sur la santé (prise de poids, sédentarité, isolement) qu’il faudra documenter et prévenir par des approches interdisciplinaires.

## Conclusion

La pandémie de COVID-19 a bouleversé le quotidien de nombreuses familles et les pratiques des organismes communautaires. De plus en plus d’études indiquent que la précarité a tendance à augmenter chez les personnes en contexte de vulnérabilité économique et sociale face à la COVID-19 et de vulnérabilité à la maladie, à la transmission communautaire et à la santé mentale. Par ailleurs, les organismes communautaires doivent respecter la distanciation sociale tout en renforçant leurs actions auprès des personnes. Avec l’habitation comme nouvelle mesure de protection liée à la COVID-19 et la nécessité d’accompagner à distance, la télésanté préventive s’est avérée une stratégie de réponse rapide aux enjeux que pose le déconfinement progressif. En période de déconfinement, la discipline et la cohésion sociale peuvent se relâcher. Au moment où la quête de libertés, la réappropriation de l’espace public et la reprise de la participation citoyenne sont recherchées, la télésanté peut jouer un rôle crucial. Accessible, facile à utiliser et de qualité, le *Programme* a renforcé les mesures préventives et de soutien et a contribué à réduire les inégalités potentielles créées par la COVID-19. Le *Programme* a engagé les familles dans un processus d’autogestion et répondu à plusieurs préoccupations et stress entraînés par la COVID-19. La télésanté peut engendrer le développement d’un lien de confiance personnalisé. Bien que ces télépratiques d’accompagnement répondent à des besoins ponctuels, elles sont innovantes : elles créent de nouvelles relations soignantes de soutien personnalisé, sont dispensées dans le « chez soi » des familles et peuvent soutenir de nouvelles formes d’intervention plus permanentes et plus efficaces. Ces mesures proactives associées à des actions concrètes dans le milieu de vie semblent favoriser l’autonomie des personnes ainsi que leur pouvoir d’action sur leur qualité de vie et leur santé. Ces éléments contribuent à plus d’équité. Quand la reconnaissance des individus, de leurs expériences et de leurs cultures coïncident avec une participation plus active au sein du milieu de vie et des processus décisionnels, nous migrons vers une approche de justice sociale plus inclusive et systémique pouvant transformer l’exercice de la citoyenneté des groupes marginalisés pour des raisons économiques ou culturelles. Les transitions rapides auxquelles nous sommes confrontées imposent un nouvel équilibre des enjeux qui exige un accompagnement ainsi que des pratiques de santé communautaire engagées et critiques. Les partenariats université-milieux constituent une avenue prioritaire à valoriser pour assurer une viabilité et une santé durable.
